# Surgical Access for Intrathecal Therapy in Spinal Muscular Atrophy with Spinal Fusion: Long-Term Outcomes of Lumbar Laminectomy

**DOI:** 10.3390/jcm14124280

**Published:** 2025-06-16

**Authors:** Tomasz Potaczek, Sławomir Duda, Jakub Adamczyk

**Affiliations:** Department of Orthopedic Surgery and Rehabilitation, Faculty of Medicine, Jagiellonian University, 34-500 Zakopane, Poland; slawomir.duda@uj.edu.pl (S.D.); jakub.adamczyk@uj.edu.pl (J.A.)

**Keywords:** spinal muscular atrophy, SMA, posterior spinal fusion, spinal fusion, lumbar laminectomy, intrathecal therapy, Nusinersen, orthopedic surgery, patient-reported outcomes

## Abstract

**Background/Objectives:** Spinal muscular atrophy (SMA) is a neuromuscular disorder frequently associated with progressive scoliosis requiring posterior spinal fusion (PSF). While Nusinersen offers significant clinical benefit, its intrathecal administration is challenging in patients with extensive spinal instrumentation and solid fusion. This study aimed to evaluate the safety, feasibility, and patient acceptance of lumbar laminectomy as a method to restore intrathecal access for repeated Nusinersen delivery in this population. **Methods**: A retrospective review was conducted in eleven patients with SMA who underwent lumbar laminectomy following prior PSF and confirmed radiographic fusion. Surgical data, injection outcomes, and patient-reported experiences were collected. A structured questionnaire assessed technical success, imaging requirements, sedation, functional response, and satisfaction. **Results**: Nine out of eleven patients (81.8%) successfully initiated intrathecal Nusinersen therapy through the laminectomy window, receiving a mean of 11.7 injections (range: 10–14). Imaging guidance was used in five cases; three required sedation or anesthesia. Intraoperative dural tears occurred in three patients and were managed without complications. Eight out of nine treated patients reported subjective motor improvement and expressed willingness to undergo the procedure again. No hardware revisions or major adverse events were observed during a mean follow-up of 48.8 months. **Conclusions**: Lumbar laminectomy is a viable and well-tolerated technique to establish intrathecal access in SMA patients with prior PSF. This approach enables sustained drug delivery and may remain clinically relevant as new intrathecal therapies emerge.

## 1. Introduction

Spinal muscular atrophy (SMA) is a rare autosomal recessive neuromuscular disorder caused by biallelic mutations in the SMN1 gene, leading to a deficiency of survival motor neuron (SMN) protein and progressive degeneration of anterior horn cells in the spinal cord [1]. The condition affects approximately 1 in 10,000 live births and manifests with symmetrical, proximal muscle weakness, hypotonia, and, in severe cases, respiratory failure [2]. SMA is clinically classified into types based on age of onset and achieved motor milestones, ranging from SMA type 1 (non-sitters) to type 3 (ambulant patients with late-onset weakness) [3].

Orthopedic complications, particularly scoliosis, are common in non-ambulatory individuals with SMA. Long thoracolumbar curves with pelvic obliquity are observed in up to 90% of SMA type 2 patients and over half of those with type 3 [4,5]. In patients with SMA type 2, the lifetime risk of requiring scoliosis surgery exceeds 80% [6]. Scoliosis in this population typically involves long C-shaped curves with pelvic obliquity and is associated with impaired sitting balance, respiratory compromise, and chronic discomfort. Surgical correction is usually performed with posterior segmental instrumentation and fusion, using constructs ranging from sublaminar wires to all-pedicle screw techniques [7,8,9]. While spinal fusion improves spinal stability and posture, it presents a significant barrier to intrathecal access.

Nusinersen, an intrathecally administered antisense oligonucleotide, has demonstrated significant motor benefits and slowed disease progression across all SMA phenotypes [10,11]. However, repetitive lumbar punctures, which are essential for maintenance dosing, are challenging in patients with prior spinal fusion, where the interlaminar spaces are obliterated, spinous processes are non-palpable, and lumbar flexion is restricted.

Alternative injection routes have been described, including transforaminal and cervical puncture and implantable intrathecal catheters [12,13,14,15]. However, these methods often require advanced imaging, general anesthesia or sedation, and may involve increased radiation exposure or procedural complexity [16,17,18,19]. Consequently, a reproducible and direct solution for intrathecal access in patients with solid fusion remains a clinical need.

In this context, lumbar laminectomy may offer a direct and reproducible surgical approach to establish intrathecal access. This procedure, widely used in spinal stenosis [20], could theoretically create a permanent anatomical “window” for repeated drug administration. However, its application specifically for restoring intrathecal access in SMA patients with prior fusion has been described only in isolated case reports and short-term procedural series [21]. Given the rarity of this indication and the evolving therapeutic landscape, there is a need for real-world data on the safety, feasibility, and patient-reported outcomes of this approach. Our study aims to fill this gap by presenting mid- to long-term outcomes in a consecutive cohort treated within a publicly funded healthcare system, where advanced access alternatives were not widely available.

## 2. Materials and Methods

### 2.1. Study Design and Setting

This retrospective analysis was based on a prospectively maintained institutional database. The study included patients treated between March 2019 and February 2020 at a single orthopedic spine surgery center, following the introduction of national reimbursement for intrathecal Nusinersen therapy. At the time of the study, no alternative administration routes or oral agents were clinically available or reimbursed in Poland.

### 2.2. Patient Selection

Inclusion criteria were: (1) genetically confirmed diagnosis of SMA, (2) history of posterior spinal fusion with instrumentation for scoliosis, (3) radiologically confirmed solid fusion of the lumbosacral spine, and (4) no feasibility of intrathecal Nusinersen administration via standard or alternative techniques. Eleven patients were enrolled: nine females and two males, with a mean age of 23.2 years (range: 10–43) at the time of laminectomy. SMA subtype classification was based on age of onset and best motor function prior to scoliosis surgery: five patients were categorized as SMA type 2a, three as 2b, and three as type 3a. Initial scoliosis correction had been performed at a mean age of 13.9 years (range: 8–27), with a mean interval of 9.4 years (range: 2–21) between spinal fusion and laminectomy. Spinal fusion constructs included Galveston rods with sublaminar wires (n = 4), hybrid constructs (pedicle screws and wires, n = 4), and all-pedicle screw systems (n = 3). At the time of treatment (2019–2020), alternative techniques such as transforaminal or cervical puncture, as well as implantable intrathecal catheter systems, were not routinely available and were not reimbursed under the national healthcare system. Therefore, laminectomy was selected as a standardized surgical approach for patients with radiographically confirmed solid spinal fusion and obliterated interlaminar spaces, in whom standard lumbar puncture was not feasible. No patients in this cohort had undergone prior unsuccessful attempts at alternative access. The decision to pursue laminectomy was based on anatomical constraints identified on preoperative imaging and the surgical team’s experience with posterior spinal approaches.

Importantly, this initiative was launched immediately after the national introduction of intrathecal Nusinersen therapy, in direct response to the needs of SMA patients who were otherwise excluded from treatment due to spinal fusion. Our guiding principle was that no patient with SMA should be denied access to available disease-modifying therapy based on technical barriers. Laminectomy was therefore offered as a pragmatic and inclusive solution to ensure access to intrathecal treatment for all eligible individuals.

### 2.3. Surgical Technique

All procedures were performed under general anesthesia with the patient in the prone position. The laminectomy level was preoperatively planned based on available imaging studies and intraoperatively confirmed with fluoroscopy. In patients with sublaminar wire instrumentation (Galveston technique), the two most caudal wire pairs were identified and exposed. The posterior fusion mass between the rods was carefully decorticated and removed using a high-speed drill and Kerrison rongeurs. Visible sublaminar wires within the laminectomy field were sectioned and extracted. The underlying dura mater was fully exposed and inspected for integrity. In cases of dural injury, primary repair with non-absorbable suture was performed, followed by application of tissue sealant. Layered closure was completed in a watertight fashion, and a standard dressing was applied. In patients with Galveston instrumentation, the main rods anchored in the iliac bones remained in place. Removal of the most caudal pairs of sublaminar wires did not compromise the stability of the construct and did not necessitate hardware revision or extension. The critical step in these cases was the careful extraction of deeply embedded sublaminar wires from within the posterior fusion mass. This maneuver, especially in the setting of limited exposure, may inadvertently lead to dural injury.

In patients with pedicle screw-based instrumentation, laminectomy was typically performed at the L4–L5 level. The rods and screw heads were exposed without hardware removal. The fusion mass was removed using the same technique as described above to access the dura through a midline window. No implants were revised or extracted during the procedure.

### 2.4. Data Collection and Outcome Measures

Clinical and surgical data were collected from operative reports and follow-up visits. Parameters included operative time, length of hospital stay, and intraoperative or postoperative complications. The mean clinical follow-up duration was 48.8 months (range: 42–53 months) following laminectomy.

A structured questionnaire was administered during follow-up visits to assess the effectiveness and feasibility of repeated intrathecal injections through the laminectomy site. In addition, we recorded whether imaging guidance or sedation was used during intrathecal injections. The decision to employ image-guided injection (CT or fluoroscopy) was based on multiple factors, including soft tissue thickness, difficulty palpating anatomical landmarks, and patient positioning limitations. Institutional factors, such as the availability of the radiology department and the presence of experienced staff, also influenced this choice. Sedation or general anesthesia was used selectively in patients with communication difficulties, anxiety, or prior distress related to procedures, and decisions were made in consultation with an anesthesiologist. The questionnaire included the following items:

1. Was intrathecal Nusinersen treatment initiated?
2. How many injections were performed?
3. Were imaging modalities (e.g., fluoroscopy, computed tomography) used for injection guidance?
4. Were sedative agents or general anesthesia required?
5. Was any subjective improvement in motor function observed?
6. Was Nusinersen therapy continued or replaced with an alternative (e.g., oral) treatment?
7. Would the patient consider undergoing the laminectomy again if needed?

## 3. Results

### 3.1. Surgical Procedure and Complications

The mean operative time for laminectomy was 70.5 min (range: 25–130 min). The average postoperative hospital stay was 4.6 days (range: 1–11 days). Intraoperative complications occurred in three patients (27.3%), all of which were incidental dural tears during the removal of sublaminar wires. Each was repaired primarily with non-absorbable sutures and sealed with tissue adhesive; no postoperative cerebrospinal fluid leakage or reoperation was required. No dural injuries were observed in patients treated with hybrid or all-pedicle screw constructs. A summary of the relationship between instrumentation type, laminectomy level, occurrence of dural tear, occurrence of delayed wound healing, and SMA type is presented in Table 1.

Delayed wound healing was observed in three cases, one of which was associated with the dural tear. A superficial surgical site infection occurred in one patient, managed successfully with oral antibiotics. No deep infections or implant-related complications were reported.

### 3.2. Intrathecal Injection Feasibility and Outcomes

Of the 11 patients who underwent laminectomy, 9 (81.8%) successfully initiated intrathecal Nusinersen therapy via the surgically created access site. Therapy was initiated a mean of 4.9 months postoperatively (range: 3–9 months), allowing time for wound healing and coordination with neurology and radiology services. Two patients did not proceed with injections: one entered a clinical trial for an oral SMN-enhancing agent, while the other deferred treatment and later transitioned to oral therapy based on evolving clinical preferences.

Among the nine patients who initiated treatment, the mean number of Nusinersen injections was 11.7 (range: 10–14) during the follow-up period. In five patients, intrathecal access was facilitated by imaging guidance (CT or fluoroscopy), typically in cases of deeper anatomical landmarks or previously documented difficulties with free-hand puncture. Institutional factors—including the availability of radiology services and skilled procedural staff—also influenced the choice of technique. The remaining four patients underwent successful free-hand injections using surface anatomical landmarks and the laminectomy site as a palpable entry point.

Sedation or general anesthesia was employed in three patients to enhance procedural tolerance, particularly in those with significant anxiety, communication challenges, or prior procedural distress. These decisions were individualized and made in consultation with anesthesiology. Eight out of nine patients (88.9%) reported subjective motor improvements during therapy. Similarly, eight patients expressed willingness to undergo the same surgical approach again if needed, reflecting overall procedural acceptability. Detailed results of the injection survey and SMA subtype classification are presented in Table 2.

## 4. Discussion

This study demonstrates that lumbar laminectomy is a feasible, effective, and relatively safe approach to enable intrathecal administration of Nusinersen in patients with spinal muscular atrophy who have undergone posterior spinal fusion. In our cohort of eleven patients, laminectomy provided durable access for repeated intrathecal injections in over 80% of cases, with a low complication rate and a high level of patient-reported satisfaction.

Progressive scoliosis is a well-documented complication of SMA, particularly in non-ambulatory individuals [4,6]. Surgical stabilization is often indicated for curves exceeding 50°, but the resulting solid posterior fusion presents a substantial obstacle to standard lumbar puncture techniques [5,7,8,9]. As the therapeutic paradigm for SMA has shifted from supportive care to disease-modifying therapies, ensuring reliable access to the intrathecal space has become a critical challenge. Nusinersen, an SMN-enhancing antisense oligonucleotide, has shown robust efficacy in improving motor outcomes and slowing disease progression [10,11], but its administration requires lifelong intrathecal access.

The choice of laminectomy in our cohort was not only a response to anatomical constraints but also a reflection of an evolving treatment paradigm in SMA care. At a time when disease-modifying therapy had just become available, there was an urgent need to ensure equitable access for all patients, including those with complex spinal anatomies following fusion. Rather than viewing these individuals as technically “ineligible,” we adopted a proactive surgical approach that aligned with the broader principle of treatment inclusion.

Several alternative techniques have been proposed for delivering Nusinersen in complex cases, including transforaminal and cervical punctures, image-guided navigation, and implantable intrathecal catheters [13,14,15,16,19,22]. While effective in select scenarios, these approaches are technically demanding, not universally available, and may expose patients to additional procedural risks or cumulative radiation [13,14]. Ko et al. [21] previously described the potential role of laminotomy in establishing access in three patients; however, concerns regarding dural injury and long-term utility remain. Other reports have described techniques such as fiducial marker placement or intraoperative fenestration to facilitate post-fusion access. A recent case study demonstrated the short-term success of a fiducial-marked laminectomy window stabilized with Gelfoam and bone wax to prevent re-fusion, although long-term outcomes were not reported [23]. Wang et al. [24] also reported on thirteen patients who underwent interlaminar fenestration at the time of scoliosis correction, achieving 1-year access success for repeated injections under ultrasound guidance. While these studies contribute to the technical toolkit, they are limited by small numbers, short durations, and a lack of longitudinal outcome data.

Our findings support the use of laminectomy as a practical alternative in carefully selected patients. The procedure was successful across various instrumentation types, including Galveston rods and pedicle screw constructs. Notably, dural tears occurred only during sublaminar wire removal and were effectively managed without adverse sequelae. Importantly, none of the patients required hardware removal, intensive care admission, or prolonged mechanical ventilation, which are key considerations in a population with often reduced pulmonary reserves.

Postoperatively, the majority of patients were able to initiate and continue intrathecal Nusinersen therapy. In several cases, free-hand injections using surface landmarks proved sufficient, obviating the need for imaging guidance. Most patients reported functional improvements and expressed willingness to repeat the procedure if necessary. These findings highlight not only the technical feasibility but also the patient-centered acceptability of this approach.

One of the distinguishing aspects of this study is the inclusion of patient-reported outcomes regarding intrathecal therapy following laminectomy. In addition to procedural success, the majority of patients reported subjective improvements in motor function, reflecting meaningful clinical benefit beyond technical feasibility. Notably, eight out of nine patients treated expressed willingness to undergo the procedure again if needed, underscoring the acceptability and perceived value of this approach. While subjective reports are inherently limited, they provide essential insights into patient satisfaction and perceived quality of life, which are increasingly recognized as important endpoints in chronic disease management. To our knowledge, few studies in this context have systematically captured patient perspectives on surgical access for Nusinersen administration.

Nevertheless, several limitations must be acknowledged. This was a single-center study with a relatively small sample size. Although the follow-up was over four years, the long-term stability of the laminectomy site for future injections requires further investigation. Moreover, our outcomes reflect a pre-risdiplam era; the increasing availability of orally administered SMN-enhancing therapies may shift patient preferences over time.

Although orally administered SMN-enhancing agents such as risdiplam are gaining popularity and may reduce the reliance on invasive procedures, the need for intrathecal access is expected to persist. Future therapeutics—including gene-modulating agents, antisense oligonucleotides, or cell-based therapies—may require direct delivery to the central nervous system. Therefore, establishing safe and reproducible surgical access routes such as lumbar laminectomy remains a relevant and forward-compatible strategy in the evolving landscape of SMA treatment.

## 5. Conclusions

This study demonstrates that lumbar laminectomy is a feasible, safe, and effective technique for restoring intrathecal access in pediatric and young adult patients with spinal muscular atrophy (SMA) who have undergone posterior spinal fusion. The procedure allowed sustained intrathecal Nusinersen therapy in the majority of cases, with a low complication rate and a high degree of patient-reported satisfaction. Importantly, no implant revision was required, and postoperative complications were minor and manageable.

Given the ongoing evolution of SMA therapeutics, safe and reproducible access to the central nervous system remains critical. Lumbar laminectomy provides a durable surgical option when standard and alternative puncture techniques are not feasible. Future studies should focus on the long-term stability of the laminectomy site and compare this approach directly with other access strategies in terms of safety, cost-effectiveness, and patient-centered outcomes.

## Figures and Tables

**Table 1:** Summary of the instrumentation type, level of laminectomy, SMA type, and intraoperative complications.

Patient	Instrumentation Type	Level of Laminectomy	Dural Tear	Delayed Wound Healing	SMA Type
Patient 1	Galvestone	L5-S1	No	Yes	3a
Patient 2	All-pedicle screw	L4-5	No	No	2b
Patient 3	Galvestone	L5-S1	Yes	Yes	2a
Patient 4	Galvestone	L5-S1	Yes	No	2a
Patient 5	Galvestone	L4-5	Yes	No	3a
Patient 6	All-pedicle screw	L4-5	No	No	2a
Patient 7	Hybrid construct	L4-5	No	Yes	2b
Patient 8	Hybrid construct	L4-5	No	No	3a
Patient 9	All-pedicle screw	L4-5	No	No	2a
Patient 10	Hybrid construct	L4-5	No	No	2a
Patient 11	Hybrid construct	L4-5	No	No	2a

**Table 2:** Detailed results of the survey.

Patient	SMA Type	Q1. Treatment Initiated	Q2. Number of Injections	Q3. Injection Guidance	Q4. Sedation Used	Q5. Motor Function Change	Q6. Therapy Status	Q7. Would Repeat Surgery
Patient 1	3a	Yes	10	CT	Sedative agents	Yes	Continues treatment	Yes
Patient 2	2b	Yes	12	Free-hand	-	Yes	Oral therapy	Yes
Patient 3	2a	Yes	12	Free-hand	-	Yes	Oral therapy	Yes
Patient 4	2a	Yes	10	CT	-	Yes	Oral therapy	Yes
Patient 5	3a	Yes	11	Free-hand	Sedative agents	Yes	Continues therapy	Yes
Patient 6	2a	Yes	13	C-arm	-	Yes	Continues therapy	Yes
Patient 7	2b	Yes	13	CT/Free-hand	General anesthesia	No change	Oral treatment	No
Patient 8	3a	Yes	11	CT	Sedative agents	Yes	Oral treatment	Yes
Patient 9	2a	Yes	14	Free-hand	-	Yes	Oral treatment	Yes
Patient 10	2a	No	0	-	-	-	Oral treatment	-
Patient 11	2a	No	0	-	-	-	Oral treatment	-

Abbreviations: SMA, spinal muscular atrophy; CT, computed tomography.

## Data Availability

The data presented in this study are available on request from the corresponding author.

## Abbreviations

The following abbreviations are used in this manuscript:

SMA	Spinal Muscular Atrophy
PSF	Posterior Spinal Fusion
CT	Computed Tomography
SMN	Survival Motor Neuron
